# Pediatric Non-Operating Room Anesthesia in Saudi Arabia: Practices, Resources, and Reported Post-Procedural Complications: A Cross-Sectional Survey

**DOI:** 10.3390/healthcare14172879

**Published:** 2026-09-07

**Authors:** Abeer A. Arab, Ahad N. Yamani, Abdulrahman Almazrooa, Alaa Sabbahi, Abdulaziz Habib, Abdulaziz Mohammed Ali Boker

**Affiliations:** 1 Department of Anesthesia and Critical Care, Faculty of Medicine, King Abdulaziz University, Jeddah 21589, Saudi Arabia; aaarab@kau.edu.sa (A.A.A.);; 2 Division of Anaesthesiology, Department of Surgery, Faculty of Medicine in Rabigh, King Abdulaziz University, Jeddah 18231, Saudi Arabia; 3 Department of Anesthesiology, Critical Care and Pain Medicine, Neuperlach Medical Center, The Munich Municipal Center, 81737 Munich, Germany; 4 College of Medicine, King Abdulaziz University, Jeddah 21589, Saudi Arabia; 5 Department of Anesthesia, Saudi German Hospital, Jeddah 21461, Saudi Arabia; 6 Skills and Clinical Simulation Center, King Abdulaziz University, Jeddah 21589, Saudi Arabia

**Keywords:** pediatric anesthesia, non-operating room anesthesia, procedural sedation, patient safety

## Abstract

**Background:** The use of pediatric non-operating room anesthesia (NORA) has increased substantially, yet information regarding current practices in Saudi Arabia remains limited. This study assessed pediatric NORA practices, resources, and reported complications among anesthesiologists in Saudi Arabia. **Methods:** A cross-sectional online survey was conducted among anesthesiologists practicing in Saudi Arabia. The questionnaire collected information on participant characteristics, indications for pediatric NORA, patient selection criteria, staffing patterns, infrastructure and equipment availability, anesthetic and monitoring practices, recovery arrangements, and post-procedural complications. **Results:** A total of 234 anesthesiologists participated. Magnetic resonance imaging (97.9%) and computed tomography (85.5%) were the most common indications. Most participants accepted patients with American Society of Anesthesiologists physical status I–III (65.8%) and reported no age restrictions (62.8%). Hospital infrastructure was considered appropriate by 85.0% of respondents. Pulse oximetry (99.6%), electrocardiography (93.2%), capnography (91.5%), and noninvasive blood pressure monitoring (89.7%) were widely used. General anesthesia (80.8%) and deep sedation (70.1%) were the most frequently used techniques. Propofol (88.9%), midazolam (82.5%), and ketamine (73.9%) were the most used sedative agents. Recovery most frequently occurred in dedicated recovery rooms (88.9%). The complications most frequently reported by respondents were delayed recovery (35.5%), agitation or delirium (27.4%), cough (21.8%), and nausea or vomiting (17.5%). **Conclusions:** Pediatric NORA in Saudi Arabia is commonly performed for imaging procedures and is generally supported by appropriate infrastructure and monitoring practices. Nevertheless, variations in resource availability highlight opportunities for further standardization and quality improvement.

## 1. Introduction

The increasing use of advanced diagnostic and therapeutic procedures has led to a substantial expansion of pediatric non-operating room anesthesia (NORA). Children frequently require anesthesia or deep sedation for procedures such as magnetic resonance imaging (MRI), computed tomography (CT), endoscopy, interventional radiology, radiation therapy, and cardiac catheterization because they are often unable to remain still or cooperate during lengthy or uncomfortable procedures. Consequently, NORA has become an integral component of contemporary pediatric anesthesia practice [1,2,3].

However, NORA presents unique challenges. Procedure rooms are often designed primarily for diagnostic or therapeutic interventions rather than anesthesia care. They may offer limited space, restricted patient access, unfamiliar equipment, and reduced availability of trained support personnel. These environmental and logistical factors may complicate airway management, patient monitoring, communication among team members, and emergency response [4]. Children represent a particularly vulnerable population because of age-related anatomical and physiological characteristics, greater susceptibility to respiratory compromise, and narrower safety margins during sedation and anesthesia. These challenges may be further compounded by the remote location of many procedures and the limited availability of immediate assistance during emergencies [5,6].

Despite these challenges, pediatric NORA can be delivered safely when appropriate systems, equipment, monitoring, and trained personnel are available. Large multicenter studies have reported low rates of serious complications and extremely rare mortality; however, adverse respiratory events such as oxygen desaturation, airway obstruction, apnea, laryngospasm, and aspiration continue to occur and require prompt recognition and intervention [7,8,9]. Consequently, several professional organizations have emphasized the importance of standardized patient assessment, appropriate patient selection, continuous monitoring, availability of resuscitation equipment, and adequate provider training for NORA [2,3,9,10,11,12].

The growing demand for pediatric NORA has also highlighted the importance of workforce preparedness and institutional resources. Previous studies have demonstrated considerable variation in sedation practices, provider training, monitoring standards, and organizational structures across institutions and countries [12,13,14]. In addition, differences in local resources, staffing models, and healthcare systems may influence the implementation of international recommendations and contribute to variability in practice [15].

In Saudi Arabia, information on pediatric NORA practices remains limited. Although the country has witnessed substantial expansion in advanced diagnostic and interventional services [16], little is known about patient selection criteria, staffing models, monitoring practices, infrastructure, recovery arrangements, and complications related to pediatric NORA. Such information is important for evaluating current practice and informing future policies, training initiatives, and safety standards. Therefore, this study aimed to assess current practices of pediatric NORA among anesthesiologists in Saudi Arabia, with particular focus on patient selection, staffing, available resources, monitoring practices, anesthetic techniques, recovery arrangements, and reported complications.

## 2. Methods

### 2.1. Study Design and Participants

This cross-sectional survey targeted anesthesiologists practicing in Saudi Arabia. Eligible participants included consultants, fellows, specialists, registrars, and residents working in anesthesia departments in governmental, military, university, National Guard, private, and specialist hospitals. Participants from specialties other than anesthesia and questionnaires with substantial missing data were excluded from the analysis. No formal a priori sample size calculation was performed. The study used convenience sampling, and the survey was distributed as widely as possible among eligible anesthesiologists during the data collection period, with the aim of obtaining the largest feasible sample.

### 2.2. Data Collection and Study Variables

Data were collected using a structured online questionnaire distributed through professional networks (official e-mails) and electronic communication platforms during April 2022. The questionnaire was developed by the study investigators based on the published literature on pediatric NORA and relevant clinical practice considerations. It was designed specifically for the present study and was not adapted from a previously validated instrument. Two anesthesiologists reviewed the questionnaire for scientific content and clarity, and minor modifications were made based on their feedback. The revised questionnaire was then pilot tested among five anesthesiologists to assess its clarity and comprehensibility before the final distribution.

The questionnaire collected information on participants’ demographic and professional characteristics, including age, sex, city of practice, and professional rank. Hospital type was classified according to administrative affiliation/healthcare sector (Ministry of Health, private, university, National Guard, military, or joint program hospital). This classification does not represent a hierarchy or level of care. Participants were asked about their experience with pediatric NORA, including the frequency of performing such procedures and the common indications. Frequency of pediatric NORA practice was assessed using the qualitative response categories “rarely,” “on demand,” and “daily.” “Rarely” was not defined according to a specific number of procedures or time interval. Indications included diagnostic and therapeutic procedures such as MRI, CT, endoscopy, radiotherapy, biopsy, angiography, digital vascular imaging, cardiac catheterization, physical examination, wound care, and other procedures.

Patient selection practices were assessed using questions on the American Society of Anesthesiologists (ASA) physical status criteria and age restrictions. The ASA physical status classification is a widely used system for assessing a patient’s preoperative health status. ASA I refers to a healthy patient without systemic disease, ASA II refers to a patient with mild systemic disease, and ASA III refers to a patient with severe systemic disease associated with functional limitations [17]. Participants were asked to indicate the highest ASA category considered eligible for pediatric NORA, with response options including ASA I only, ASA I–II, ASA I–III, or no specific ASA-based eligibility criterion. Participants were also asked whether they excluded neonates, infants, or children younger than five years from such procedures. Staffing patterns were evaluated by asking participants about other healthcare professionals involved during pediatric NORA, including anesthesia technicians, anesthesia consultants, anesthesia specialists, anesthesia residents, and anesthesia nurses.

The questionnaire also assessed infrastructure and resource availability. Participants were asked whether their hospital setup was appropriate for providing pediatric NORA, whether procedure rooms were sufficiently large and safe, and whether equipment checklists were available before procedures. “Appropriate” reflected the respondent’s overall clinical assessment of the hospital setup and was not defined according to predetermined structural or equipment criteria. Similarly, “sufficiently large” reflected the respondent’s assessment of whether the available space was adequate for the procedure, without specifying a minimum room area or other numerical threshold. Specific aspects of infrastructure and resource availability were assessed separately. Information was collected regarding the availability of essential equipment, including oxygen sources, monitors, pulse oximeters, suction apparatuses, anesthesia devices, emergency care bags, emergency carts, defibrillators, and fixed-line telephones.

Anesthetic practices were assessed through questions regarding pre-procedural fasting assessment, use of premedication, preferred anesthetic techniques, regional anesthesia practices, monitoring methods, sedative agents, antagonism practices, and antagonist agents. Anesthetic techniques included mild sedation, moderate sedation, deep sedation, and general anesthesia. These categories were presented as clinical response options without additional operational definitions, and participants classified their practice according to their clinical understanding of these terms. Regional anesthesia techniques included caudal anesthesia, peripheral nerve blockade, and spinal anesthesia. Monitoring practices included the use of pulse oximetry, electrocardiography (ECG), capnography, and noninvasive blood pressure monitoring. Sedative agents included propofol, midazolam, ketamine, dexmedetomidine, and other agents. Participants were also asked about the frequency of antagonist use and the types of antagonists commonly used, including naloxone, neostigmine, sugammadex, and flumazenil.

Recovery practices were assessed by asking participants about the usual location of recovery following the procedure, including recovery rooms, procedure rooms, wards, and waiting areas. Finally, participants were asked to identify the post-procedural complications they encountered most frequently during pediatric NORA. These complications included delayed recovery, agitation or delirium, cough, nausea or vomiting, desaturation, airway obstruction, bradyarrhythmias, and other complications. No study-specific numerical or diagnostic criteria were provided for these complications. In particular, no predefined duration was specified for delayed recovery, no pulse oximetry cut-off was specified for desaturation, and no predefined diagnostic criteria were provided for airway obstruction or bradyarrhythmia. These responses therefore reflected participants’ clinical interpretation and experience. The “other” category allowed participants to report complications not included among the prespecified response options.

### 2.3. Statistical Analysis

Data were analyzed using IBM SPSS Statistics version 22 (IBM Corp., Armonk, NY, USA). Age was presented as mean ± standard deviation (SD). Categorical variables were presented as frequencies and percentages. Comparisons between consultants and non-consultants in the supplementary analyses were performed using the chi-square test or Fisher’s exact test when appropriate. A two-sided *p*-value < 0.05 was considered statistically significant.

## 3. Results

A total of 234 anesthesiologists participated in this study. Their mean age was 43.8 ± 8.6 years, and most participants were male (79.9%). The most common workplace cities were Jeddah (36.7%), Riyadh (27.3%), and Dammam (18.8%). Participants worked mainly in Ministry of Health hospitals (31.2%), private hospitals (18.4%), university hospitals (17.1%), National Guard hospitals (15.8%), and military hospitals (15.8%). More than half of the participants were consultants (56.8%) (Table 1).

Regarding pediatric NORA, 86.8% of participants reported performing these procedures on demand, 8.5% reported doing them rarely, and 4.7% reported doing them daily. MRI was the most common indication (97.9%), followed by CT (85.5%), endoscopy (61.5%), radiotherapy (47.0%), and biopsy (42.3%). Most participants considered patients with ASA I–III suitable candidates (65.8%), while 23.1% accepted only ASA I–II patients. Regarding age restrictions, 62.8% reported no restrictions, whereas 23.5% excluded neonatal-age patients. The most reported team member assisting during these procedures was an anesthesia technician (97.0%) (Table 2).

Most participants considered their hospital setup appropriate for providing pediatric NORA (85.0%). In addition, 66.7% reported that the procedure room was sufficiently large, and 87.6% considered it safe. Oxygen sources were available in all settings. Other commonly available equipment included monitors (97.0%), pulse oximeters (94.9%), suction apparatuses (91.9%), emergency care bags (91.0%), and anesthesia devices (89.3%). Defibrillators were available in 51.7% of settings. Equipment checklists before procedures were available in 65.4% of institutions (Table 3).

All participants reported assessing fasting status before anesthesia. Premedication on the day before anesthesia was reported by 6.8% of participants. General anesthesia was the most frequently used anesthetic method (80.8%), followed by deep sedation (70.1%), moderate sedation (55.6%), and mild sedation (35.9%). Most participants did not use regional anesthesia techniques (80.3%). Pulse oximetry (99.6%), ECG (93.2%), capnography (91.5%), and noninvasive blood pressure monitoring (89.7%) were commonly used. The most frequently used sedative agents were propofol (88.9%), midazolam (82.5%), ketamine (73.9%), and dexmedetomidine (31.6%). Antagonists were sometimes used by 59.8% of participants and rarely used by 32.5%. Naloxone was the most used antagonist agent (49.6%) (Table 4).

Recovery most commonly took place in a recovery room (88.9%), followed by the same procedure room (33.3%) and waiting room (18.4%). The most frequently reported post-procedural complication was delayed recovery (35.5%), followed by agitation/delirium (27.4%), cough (21.8%), nausea or vomiting (17.5%), and desaturation (10.7%). Airway obstruction (8.1%), bradyarrhythmia (6.8%), and other complications (5.6%) were less frequently reported (Table 5).

In supplementary analyses, consultants reported more frequent use of ketamine and dexmedetomidine than non-consultants (*p*-value < 0.05) (Supplementary Table S1). Non-consultants reported cough more frequently than consultants (*p*-value < 0.05), while no other notable differences in reported complications were observed between the two groups (Supplementary Table S2).

## 4. Discussion

This national survey provides an overview of current pediatric NORA practices in Saudi Arabia. Most respondents reported performing pediatric NORA for imaging procedures, particularly MRI and CT, and commonly accepted patients with ASA physical status I–III. General anesthesia and deep sedation were the predominant approaches, supported by widespread use of standard monitoring modalities and recovery facilities. Although most participants reported adequate availability of infrastructure and equipment, delayed recovery and emergence-related complications were among the complications most frequently reported by respondents.

MRI and CT were the most common indications for pediatric NORA in the present study. This finding is consistent with previous reports showing that imaging procedures account for a substantial proportion of pediatric NORA because they often require prolonged immobility and cooperation that may not be achievable in young children without sedation or anesthesia [1,3,18,19,20,21,22,23].

Most participants accepted patients with ASA physical status I–III, although variation was observed in both ASA-based and age-based eligibility criteria. Such variation may reflect differences in institutional policies, available resources, provider experience, and the availability of pediatric-specific support services. Unlike operating room settings, NORA procedures are often performed in remote locations where access to additional personnel, equipment, and emergency resources may be more limited [6]. As a result, some providers may adopt more restrictive eligibility criteria, particularly for younger children or those with significant comorbidities. Children are not simply small adults; neonates, infants, and older children differ in anatomy, physiology, pharmacology, body composition, airway characteristics, fluid needs, and drug metabolism. Neonates are particularly vulnerable because of immature hepatic/renal function, underdeveloped blood–brain barrier, altered protein binding, and high sensitivity to respiratory and cardiovascular drug effects [6]. Nearly one-quarter of participants reported excluding neonates from pediatric NORA, which may reflect concerns regarding the physiological vulnerability of this age group. Previous studies have reported higher rates of adverse events among younger children [24,25], which may partly explain the more cautious eligibility criteria reported by some participants in the present study.

Most participants reported the availability of appropriate facilities, monitoring equipment, and recovery arrangements for pediatric NORA. Nevertheless, equipment checklists and defibrillators were not universally available. International recommendations emphasize the importance of adequate space, monitoring, emergency equipment, and trained personnel when providing anesthesia outside the operating room [4,10,18,26]. The widespread use of pulse oximetry, ECG, capnography, and noninvasive blood pressure monitoring observed in our study is consistent with these recommendations and suggests a high level of awareness regarding patient safety during pediatric NORA. Nevertheless, the availability of defibrillators in only about half of institutions and the absence of equipment checklists in a substantial proportion of settings suggest important gaps in emergency preparedness. These findings highlight the need for standardized minimum equipment requirements and routine pre-procedure safety checks to ensure consistent preparedness for pediatric NORA across healthcare facilities in Saudi Arabia.

Delayed recovery and emergence agitation were the complications most frequently reported by respondents, whereas serious cardiorespiratory complications were reported less frequently. These findings should not be interpreted as procedure-level incidence rates because respondents reported whether they had encountered each complication rather than the number of events among a defined number of pediatric NORA procedures. Therefore, direct numerical comparison with complication rates reported in prospective registries is not appropriate. Nevertheless, large pediatric registries provide useful context regarding the types and severity of adverse events occurring during pediatric sedation and anesthesia outside the operating room.

Previous large pediatric registries have generally reported low rates of major adverse events. In a multicenter Pediatric Sedation Research Consortium study of 30,037 sedation encounters, no deaths were reported, and cardiopulmonary resuscitation was required only once; however, oxygen desaturation occurred in 157 per 10,000 sedations, while laryngospasm and stridor each occurred in 4.3 per 10,000 sedations [7]. Similarly, Grunwell et al. reported an overall adverse event rate of 7.26% and a severe adverse event rate of 1.77% among 22,645 children undergoing ketamine sedation outside the operating room [8]. In addition, Lee et al. found respiratory adverse events in 3.9% of children undergoing propofol-based sedation for imaging procedures, particularly among those with cardiac or neurological comorbidities and a history of upper respiratory problems [9]. Of note, the relatively low frequency of severe complications reported in our study should be interpreted cautiously because these findings were based on clinicians’ recollection rather than prospectively collected outcome data.

The variation observed in pediatric NORA practices in the present study suggests that these practices are not standardized across Saudi Arabia. Differences were identified in patient selection criteria, age restrictions, staffing models, availability of emergency equipment, and recovery arrangements. For example, not all respondents reported the availability of equipment checklists or defibrillators, and considerable variation was observed in the acceptance of neonates and higher-risk patients. These findings suggest that institutions may rely on local policies and individual experience when making decisions regarding pediatric NORA. In this context, nationally developed recommendations may help promote greater consistency in areas such as patient assessment, monitoring, staffing requirements, equipment availability, and recovery practices. Such recommendations should be based on local data and developed with input from relevant stakeholders to ensure that they are practical and applicable across different healthcare settings in Saudi Arabia.

This study provides one of the few available descriptions of pediatric non-operating room anesthesia practices in Saudi Arabia. However, several limitations should be acknowledged. First, the study relied on self-reported responses, which may not fully reflect actual clinical practice. Second, the survey was based on a convenience sample of anesthesiologists, raising the possibility of selection bias, as anesthesiologists with a greater interest in pediatric NORA may have been more likely to participate. In addition, because the survey was distributed through professional networks and electronic communication platforms, the number of individuals who received the invitation could not be determined, and a response rate could not be calculated. As a consequence, the sample cannot be considered fully representative of all anesthesiologists practicing in Saudi Arabia. Third, responses were obtained from individual anesthesiologists rather than institutions, and the number of participants varied across hospitals and regions. This may have influenced the overall estimates of practice patterns. Fourth, the reported complications were based on participants’ recollection and were not linked to specific procedures, patient characteristics, anesthetic techniques, or objectively recorded clinical outcomes. Fifth, the survey did not collect information on pediatric NORA case volume, adherence to specific guidelines, factors that may influence practice patterns, and perceived complication rates. Sixth, the survey design did not allow exploration of the reasons underlying participants’ practices and preferences. Future qualitative research may help explore institutional, organizational, and professional factors underlying the variations in practice observed in the present study. Seventh, data were collected in April 2022. Pediatric NORA practices, available technologies, monitoring standards, and institutional policies may have evolved since data collection; therefore, the findings may not fully reflect contemporary practice. Eighth, several questionnaire items relied on respondents’ clinical judgment and were not accompanied by standardized operational definitions or numerical thresholds. Terms such as “rarely,” “appropriate,” “sufficiently large,” and “safe,” as well as categories of sedation and reported complications, may therefore have been interpreted differently by participants, introducing some degree of measurement variability. In particular, the reported complications reflect respondents’ clinical experience rather than events identified using standardized diagnostic criteria or procedure-level incidence data.

## 5. Conclusions

The present study provides insight into pediatric NORA practices in Saudi Arabia. The findings suggest that pediatric NORA is integrated into routine anesthetic practice across different healthcare settings and is supported by established approaches to patient assessment, monitoring, and recovery. At the same time, variations in patient selection criteria, equipment availability, infrastructure, and peri-procedural practices were observed. These findings highlight the need for national guidelines to standardize infrastructure requirements, equipment availability, safety protocols, and clinical practices for pediatric NORA across healthcare facilities. Future studies should evaluate the relationship between these practice variations and clinical outcomes, including respiratory and recovery-related complications, assess adherence to international recommendations, and identify barriers and facilitators to implementing standardized pediatric NORA practices in Saudi Arabia.

## Figures and Tables

**Table 1:** Participant characteristics (n = 234).

Variables		Frequency	%
Age (years) mean ± SD		43.8 ± 8.6	
Sex	Men	187	79.9
	Women	47	20.1
Workplace city	Jeddah	86	36.7
	Riyadh	64	27.3
	Dammam	44	18.8
	Makkah	14	6.0
	Taif	7	3.0
	Hail	5	2.1
	Al Medina	4	1.7
	Qatif	2	0.9
	Jizan	2	0.9
	Dhahran	2	0.9
	Al Ahsa’a	2	0.9
	Jubail	1	0.4
	Al Qassim	1	0.4
Hospital sector	Ministry of Health Hospital	73	31.2
	Private Hospital	43	18.4
	University Hospital	40	17.1
	National Guard Hospital	37	15.8
	Military Hospital	37	15.8
	Joint Program	4	1.7
Rank	Professor	1	0.4
	Consultant	133	56.8
	Fellow	3	1.3
	Senior specialist/Senior registrar	36	15.4
	Specialist/Registrar	37	15.8
	Resident	24	10.3

Data are presented as frequency and %. Age is presented as mean ± SD.

**Table 2:** Clinical indications, patient selection criteria, and staffing pattern (n = 234).

Variables		Frequency	%
Frequency of practice	Rarely	20	8.5
	On demand	203	86.8
	Daily	11	4.7
Indications *	MRI	229	97.9
	CT	200	85.5
	Endoscopy	144	61.5
	Radiotherapy	110	47.0
	Biopsy	99	42.3
	Angiography	76	32.5
	Digital vascular imaging	59	25.2
	Physical examination	28	12.0
	Wound care	26	11.1
	Cardiac catheterization	18	7.7
	Others	46	19.7
American Society of Anesthesiologists (ASA) criteria for candidacy	ASA I	3	1.3
	ASA I-II	54	23.1
	ASA I-III	154	65.8
	None	23	9.8
Age restrictions	Exclusion of neonatal age	55	23.5
	Exclusion of infantile age	24	10.3
	Exclusion of less than 5 years	8	3.4
	None	147	62.8
Other team members *	Anesthesia technician	227	97.0
	Anesthesia consultant	18	7.7
	Anesthesia resident	10	4.3
	Anesthesia nurse	10	4.3
	Anesthesia specialist	7	3.0

* Multiple response question.

**Table 3:** Infrastructure and equipment availability (n = 234).

Variables		Frequency	%
Is the hospital setup appropriate for providing pediatric anesthesia outside the operating room?	No	35	15.0
	Yes	199	85.0
Is the room sufficiently large for the procedure?	No	78	33.3
	Yes	156	66.7
Is the room safe for the procedure?	No	29	12.4
	Yes	205	87.6
Available equipment *	Oxygen source	234	100.0
	Monitor	227	97.0
	Pulse oximetry	222	94.9
	Suction apparatus	215	91.9
	Emergency care bag	213	91.0
	Anesthesia device	209	89.3
	Emergency cart	178	76.1
	Fixed line telephone	153	65.4
	Defibrillator	121	51.7%
Equipment checklist before the procedure	Available	153	65.4
	Available but not required	42	17.9
	Not available	39	16.7

* Multiple response question.

**Table 4:** Anesthesia/sedation methods reported as frequently used (n = 234).

Variables		Frequency	%
Do you question the fasting period?	No	0	0.0
	Yes	234	100.0
Do you use pre-medication the day before anesthesia	No	218	93.2
	Yes	16	6.8
Frequently used method for anesthesia *	Mild sedation	84	35.9
	Moderate sedation	130	55.6
	Deep sedation	164	70.1
	General anesthesia	189	80.8
Most used regional anesthesia	Caudal anesthesia	17	7.3
	Peripheral blockade	6	2.6
	Spinal anesthesia	3	1.3
	All of them	20	8.5
	None of them	188	80.3
Frequently used method for non-invasive monitoring *	Pulse oximetry	233	99.6
	ECG	218	93.2
	Capnography	214	91.5
	Noninvasive blood pressure	210	89.7
Frequently used hypnotic agents for sedation *	Propofol	208	88.9
	Midazolam	193	82.5
	Ketamine	173	73.9
	Dexmedetomidine	74	31.6
	Others	19	8.1
Use of antagonism	Rarely	76	32.5
	Sometimes	140	59.8
	Always	18	7.7
Frequently used antagonist agent *	Naloxone	116	49.6
	Neostigmine	98	41.9
	Sugammadex	89	38.0
	Flumazenil	87	37.2

* Multiple response question.

**Table 5:** Recovery practices and complications (n = 234).

Variables		Frequency	%
Place of recovery *	Recovery room	208	88.9
	Same room	78	33.3
	Waiting room	43	18.4
	Ward	4	1.7
Post-procedural complications reported by respondents *	Delayed recovery	83	35.5
	Agitation/delirium	64	27.4
	Cough	51	21.8
	Nausea or vomiting	41	17.5
	Desaturation	25	10.7
	Airway obstruction	19	8.1
	Bradyarrhythmia	16	6.8
	Others	13	5.6

* Multiple response question. Percentages represent the proportion of respondents reporting each complication and should not be interpreted as complication incidence per procedure.

## Data Availability

The dataset generated and analyzed during the current study is not publicly available due to ethical and administrative restrictions. However, the corresponding author can provide the data upon reasonable request.

## Supplementary Materials

The following supporting information can be downloaded at: https://www.mdpi.com/article/10.3390/healthcare14172879/s1, Table S1: Comparison between consultants (n = 133) and non-consultants (n = 97) regarding selected practices. Table S2: Comparison between consultants (n = 133) and non-consultants (n = 97) regarding complications.
