# Supplementary material for: Pediatric Non-Operating Room Anesthesia in Saudi Arabia: Practices, Resources, and Reported Post-Procedural Complications: A Cross-Sectional Survey
Source: Healthcare (Basel). 2026 Sep 7;14(17):2879. doi: 10.3390/healthcare14172879 (PMC13565593; doi:10.3390/healthcare14172879)
Supplement: Supplementary file 1 [file healthcare-14-02879-s001.zip › healthcare-4463848-supplementary.pdf]

Title: Pediatric Non-Operating Room Anesthesia in Saudi Arabia: Current Practices, Resources, and Post-Procedural Complications

Supplementary Table S1: Comparison between consultants (n = 133) and non-consultants (n = 97) regarding selected practices

Variables		Consultant n (%)	Non-consultant n (%)
Frequently used method for anesthesia	Mild sedation	45 (33.8)	38 (39.2)
	Moderate sedation	76 (57.1)	54 (55.7)
	Deep sedation	97 (72.9)	64 (66.0)
	General anesthesia	110 (82.7)	75 (77.3)
Frequently used regional anesthesia	Caudal anesthesia	8 (6.0)	9 (9.3)
	Peripheral blockade	3 (2.3)	3 (3.1)
	Spinal anesthesia	2 (1.5)	1 (1.0)
	All of them	12 (9.0)	8 (8.2)
	None of them	108 (81.2)	76 (78.4)
Hypnotic agents used for sedation	Propofol	119 (89.5)	86 (88.7)
	Midazolam	111 (83.5)	80 (82.5)
	Ketamine	107 (80.5)	62 (63.9)*
	Dexmedetomidine	49 (36.8)	23 (23.7)*
Use of antagonism	Rarely	47 (35.3)	28 (28.9)
	Sometimes	76 (57.1)	61 (62.9)
	Always	10 (7.5)	8 (8.2)

*P-value < 0.05

Supplementary Table S2: Comparison between consultants (n = 133) and non-consultants (n = 97) regarding complications

Variables		Consultant n (%)	Non-consultant n (%)
Frequently encountered post-procedural complications	Delayed recovery	42 (31.6)	39 (40.2)
	Agitation/delirium	35 (26.3)	26 (26.8)
	Cough	20 (15.0)	31 (32.0)*
	Nausea or vomiting	23 (17.3)	17 (17.5)
	Desaturation	15 (11.3)	9 (9.3)
	Airway obstruction	11 (8.3)	7 (7.2)
	Bradyarrhythmia	9 (6.8)	6 (6.2)
	Others	5 (3.8)	7 (7.2)

*P-value < 0.05
